# Liver Transplantation from a Donor with Multiple Biliary Hamartomata

**Published:** 2013-02-01

**Authors:** R. F. Saidi, V. Yoon, N. Jabbour, S. A. Shah, A. Bozorgzadeh

**Affiliations:** *Division of Organ Transplantation, Department of Surgery, University of Massachusetts Medical School, Worcester, MA, USA*

**Keywords:** Liver transplantation, Biliary hamartoma, Liver, benign lesions

## Abstract

Biliary hamartomata are rare benign lesions. Herein, we report on a 48-year-old man with a history of end-stage liver disease secondary to alcoholic liver disease. The patient received an orthotropic liver transplant from a brain-death woman. At the time of recovery, there were multiple lesions in the transplanted liver measuring 7–10 mm. Pathology revealed multiple biliary hamartomata. The postoperative course of the recipient was uncomplicated and he was discharged home 10 days after the transplantation.

## INTRODUCTION

Biliary hamartomata are rare benign lesions which were first described in 1918 by von Meyenburg [1]. Typically, these biliary malformations form cystic structures of various sizes within an array of architectural distortion [1, 2]. These lesions have been shown to mimic malignant tumors, making them difficult to diagnose [1-3].

## CASE REPORT

**Figure 1:**
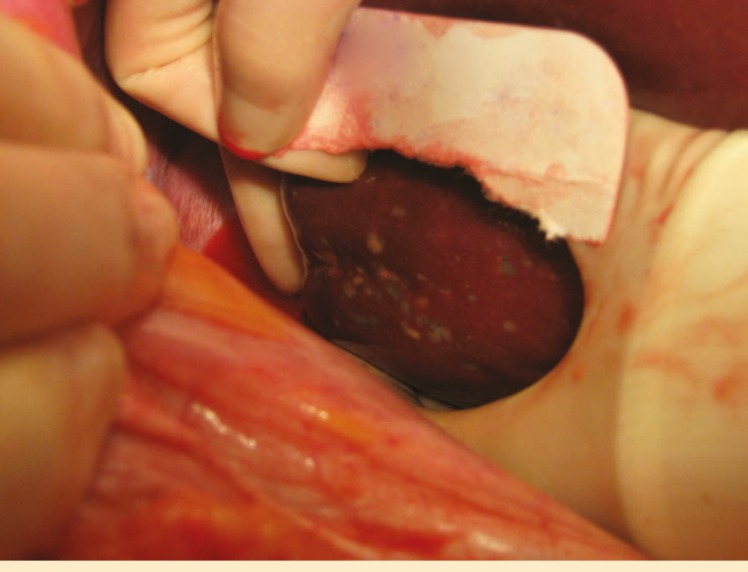
Multiple hepatic lesions on exploration turned out to be biliary hamartoma on biopsy.

Herein, we report on a 48-year-old man with a history of end-stage liver disease secondary to alcoholic liver disease with signs of decompensation manifested by massive ascites requiring multiple bouts of large volume paracentesis, and hepatic hydrothorax requiring thoracentesis. The patient also had an upper endoscopy which showed a diffuse portal hypertensive gastropathy. He was listed for liver transplantation with MELD of 15. He eventually, received an orthotropic liver transplant with piggyback technique. The donor was a 49-year-old woman with history of hypertension who became brain-death secondary to anoxic encephalopathy due to massive pulmonary embolism and cardiac arrest. Serum AST and ALT were initially 111 and 121 (IU/L), but rose to mid 400 before recovery. At the time of recovery, there were multiple lesions in the liver measuring 7–10 mm (Fig 1). Pathology revealed multiple biliary hamartomata (von Meyenburg complex). His postoperative course was uncomplicated and he was discharged home 10 days after the transplantation. He had an episode of raise in transaminases and was diagnosed with acute cellular rejection on biopsy. He responded well to steroid pulse therapy. Currently, the patient is doing very well two years after liver transplantation.

## DISCUSSION

Biliary hamartomata are rare benign tumors of the liver that exhibit abnormal histological features, such as ductules that contain a single layer of cuboidal epithelium surrounded by a fibrous stroma. The pathogenesis of these lesions is the result of arrested development and remodeling of the small, peripheral intrahepatic biliary ducts [2]. Biliary hamartomata are thought to reside on the spectrum of congenital hepatic fibrosis and may be associated with other congenital diseases, including hepatic fibrosis, polycystic kidney disease, and Caroli's disease [2]. Grossly, these lesions are usually multiple (von Meyenburg complex) and may be dispersed throughout the liver. Macroscopically, biliary hamartomata are whitish-gray and range from 5–10 mm in diameter [1, 2]. The bile ducts within these lesions are also variable, ranging from narrow to very dilated [1, 2]. Although the risk of malignant conversion of biliary hamartoma is low, malignant transformation to cholangiocarcinoma has been reported [4].

The variable reported incidence of these macroscopic lesions adds to the diagnostic confusion. The reported incidence in autopsy series ranges from 0.69% in cases of macroscopic examination to 2.8% in microscopic surveys [1, 2]. While histological analysis provides the most accurate diagnosis, ultrasonography, CT, and MRI may also show these lesions. The definitive diagnosis of biliary hamartomata should solely be based on tissue sampling.

The greatest challenge facing the field of organ transplantation today is increasing the number of allografts available for transplantation. Increasing utilization of marginal organs has been advocated to address the organ shortage. To date, there have been very few reports on the use of donor livers with this finding [5]. We suspect that the natural history of these lesions in the transplant recipient will be benign as it usually is in the host.

## References

[1] Mortele B, Mortele K, Seynaeve P (2002). Hepatic bile duct hamartomas (von Meyenburg complexes): MR and MR cholangiography findings. J Comput Assist Tomogr.

[2] Principe A, Lugaresi ML, Lords RC (1997). Bile duct hamartomas: diagnostic problems and treatment. Hepatogastroenterology.

[3] Cheung YC, Tan CF, Wan YL (1997). MRI of multiple biliary hamartomas. Br J Radiol.

[4] Orii T, Ohkohchi N, Sasaki K (2003). Cholangiocarcinoma arising from preexisting biliary hamartoma of liver—report of a case. Hepatogastroenterology.

[5] Guarrera JV, Alkofer BJ, Feirt N (2007). Discovery of diffuse biliary microhamartomas during liver procurement. Liver Transpl.

